# The effect of beta-adrenergic blockade on inflammatory and cardiovascular responses to acute mental stress

**DOI:** 10.1016/j.bbi.2018.03.027

**Published:** 2018-05

**Authors:** Andrew Steptoe, Amy Ronaldson, Karen Kostich, Antonio I. Lazzarino, Livia Urbanova, Livia A. Carvalho

**Affiliations:** Department of Epidemiology and Public Health, University College London, London WC1E 6BT, UK

**Keywords:** Inflammation, Stress, Sympathetic nervous system, Sex differences, Blood pressure, Pharmacological blockade

## Abstract

Acute mental stress elicits increases in plasma cytokine concentrations in humans, but the underlying mechanisms remain poorly understood. We assessed the impact of beta-adrenergic blockade on plasma interleukin 6 (IL-6) and IL-1 receptor antagonist (IL-1Ra) responses in a parallel group, double-blind randomised placebo-controlled trial involving 64 healthy young adult volunteers. Participants were administered 80 mg slow-release propranolol or placebo daily for 7 days before the stress testing session in which responses to 3 behavioural challenges (public speaking, mirror tracing, mental arithmetic) were evaluated. Propranolol administration was associated with reduced baseline levels of heart rate and IL-1Ra, and systolic blood pressure (BP) in men. Tasks stimulated increased plasma IL-6 concentrations sampled 45 min and 75 min after challenge, but these responses were blocked by propranolol in men (*p* < 0.001). Propranolol did not influence IL-6 responses in women, or IL-1Ra in either sex. Blood pressure and heart rate increased markedly during the tasks, but there was no differential stress reactivity in propranolol and placebo conditions. The results of the study support a role of sympathetic nervous system activation in stimulating acute IL-6 responses to stress, but only in men. The reasons for the differences between men and women remain to be resolved.

## Highlights

• The mechanisms underlying acute stress-induced IL-6 responses are poorly understood.
• We assessed the effects of beta-blockade with propranolol in a double-blind study.
• Beta-blockade reduced IL-6 responses to stress in men but not women.
• Blood pressure and heart rate responses was not affected by propranolol.
• The study provides partial support for SNS involvement in stress-induced inflammation.

## 1 Introduction

Inflammation is involved in a range of serious health problems including coronary heart disease (CHD), some cancers, chronic pain, and depression (Elinav et al., 2013, Hansson and Hermansson, 2011, Louati and Berenbaum, 2015, Miller and Raison, 2016). Psychosocial factors such as early life trauma, low socioeconomic status (SES), caregiver strain and other adult stressors have also been associated with low-grade systemic inflammation (Danese and Lewis, 2017, Kiecolt-Glaser et al., 2015, Rohleder, 2014, Stringhini et al., 2013). This has led to the conjecture that inflammation mediates in part the association between psychosocial adversity and health outcomes. This link is supported by experimental studies demonstrating that acute psychological stress stimulates increased concentration of circulating inflammatory markers, notably interleukin 6 (IL-6) but also IL-1β and tumor necrosis factor alpha (TNFα) (Marsland et al., 2017, Steptoe et al., 2007). Additionally, individual differences in psychosocial factors such as loneliness and hostility appear to modulate the magnitude of inflammatory responses to acute psychological stress (Hackett et al., 2012, Hackett et al., 2015).

The biological mechanisms underlying inflammatory responses to acute stress are only partly understood. Psychological stress elicits rapid increases in expression of nuclear factor κB (NF- κB), a transcription factor promoting the production of IL-6 and IL-1β (Bierhaus et al., 2003, Kuebler et al., 2015). Increases in IL-1β and IL-6 mRNA expression from leukocytes have also been described (Brydon et al., 2005, Kuebler et al., 2015, McInnis et al., 2015). Other factors that may be relevant include redistribution of circulating white blood cell subpopulations that expression proinflammatory cytokines, and release of lymphocytes from marginal pools (Steptoe et al., 2007). Research in animal models strongly implicates sympathetic nervous system activation in these responses (Bierhaus et al., 2003, Sanders and Kavelaars, 2007). In humans, positive correlations between plasma IL-6 responses to acute stress and cardiovascular activity have also been observed, again suggestive of sympathetic nervous system involvement (Brydon et al., 2005, Kop et al., 2008).

Another approach to investigating the pathways underlying acute inflammatory responses involves pharmacological blockade (Van Hedger et al., 2017). Rodent studies indicate that beta-adrenergic blockade inhibits stress-induced increases in inflammation (Hanke et al., 2012, Powell et al., 2013), but evidence from humans is inconclusive. The most detailed study was a double-blind analysis of 64 healthy middle-aged men and women randomised to daily 80 mg propranolol, 100 mg aspirin or placebo for 5 days (von Kanel et al., 2008). There was no effect of propranolol on plasma IL-6 responses up to 105 min after the Trier Social Stress Test (TSST), while aspirin attenuated stress-induced increases. The explanation for the lack of effect of propranolol is not clear. Rohleder (2008) suggested that the timing of stress testing might have been relevant, but it is difficult to know what would have been more appropriate. In an attempt to understand these processes better, we therefore carried out a parallel group double-blind trial evaluating the effect of propranolol vs placebo on IL-6 and cardiovascular responses to acute psychological stress in healthy young adults. We did not use the TSST but a similar battery of behavioural challenges, and measured IL-1Ra as an additional inflammatory biomarker. We hypothesised that 7 days of 80 mg propranolol would block stress-induced increases in inflammation and reduce systolic blood pressure (BP) and heart rate stress reactivity.

## 2 Method

### 2.1 Participants

Participants were 69 healthy volunteers recruited from the UCL campus for a study assessing the effects of pharmacological probes on stress responsivity. All data were collected with the written informed consent of the participants and ethical approval was obtained from the UCL Research Ethics Committee. Participants were aged 18 years and over, reportedly in good health, and not taking medications regularly (excluding the contraceptive pill). Exclusion criteria included haematological, pulmonary, liver, renal, gastrointestinal, heart, cerebrovascular, and psychiatric disease, history of thromboembolism, and participants were free of current infection. Individuals suffering from asthma, who had known allergies to the study medications, previous gastrointestinal bleedings, or who were currently pregnant or breastfeeding were excluded. Only those with BP in the normal range were included (90/60 mmHg–140/90 mmHg). Three people failed to complete the study, one dropped out because of side effects, and one was excluded after taking cold medication, leaving 64 in the final sample (20 men, 44 women). Volunteers were paid a small honorarium at the end of the study. The study was approved by the UCL Research Ethics Committee, and all participants gave signed informed consent.

### 2.2 Treatment conditions

Participants were randomised to propranolol or placebo conditions stratified by sex to ensure equal numbers of men and women in each group. None of the researchers involved in the study were aware of the treatment condition to which individuals were assigned. Propranolol is a non-selective beta-blocker, inhibiting the effects of catecholamines on both β1- and β2-adrenoceptors. Participants were administered 80 mg of sustained-release propranolol or identical placebo once a day after breakfast for 7 days. The dose was selected as the minimum recommended clinical dose for the treatment of hypertension, and was therefore deemed appropriate for healthy volunteers in order to minimise the likelihood of side effects.

### 2.3 Measures and procedure

Participants attended a brief session in the laboratory at which body composition was measured and a questionnaire containing demographic and psychosocial measures was completed. They received a bottle containing 12 pills of the study medication and were instructed to take one capsule every morning after breakfast for the following 7 days. Participants were advised not to take any other medications or herbal remedies while taking part in the study and to avoid alcohol and vigorous physical activity.

Study participants returned 7 days later for the laboratory stress session either in the morning (9:00 h) or afternoon (13:30 h), bringing back the pill bottle so that the remaining capsules could be counted. They were instructed not to exercise before the session, not to drink alcohol the evening before, and not to consume any caffeine on the morning of the testing day. They were asked to eat a light breakfast and/or lunch. Anxiety and positive affect over the past week were assessed. Then a Portapres-2 (Finapres Medical Systems, Amsterdam, NL) was fitted for the continuous monitoring of BP and heart rate, and an intravenous cannula was inserted for blood sample collection. The participant subsequently rested quietly for 30 min, followed by the baseline blood draw. Participants also rated subjective stress.

A behavioural task battery consisting of three tasks was then administered. First was a socially evaluative public speaking task as previously used by our group (Ghiadoni et al., 2000). The participant was asked to imagine a situation in which they had been falsely accused of shoplifting. They were required to prepare a statement in their defence for 2 min and to present it for 3 min. They were seated facing a video camera, and were told that images would be analysed and rated for fluency and competence. Second was a mirror tracing task used extensively in psychophysiological research (Matthews et al., 2003, Steptoe et al., 2002). The participant traced around a star seen in mirror image with a metal stylus for 5 min. Errors were indicated by a loud sound. The participant was instructed to trace around the star as many times as possible in 5 min while making minimal errors. Third was a serial subtraction task, in which the participant serially subtracted the number 13 from 1,022 as fast and as accurately as possible for 5 min (Kirschbaum et al., 1993). After every failure, the participant had to restart at 1,022. Blood pressure and heart rate monitoring continued throughout, and a second blood sample was drawn immediately after tasks. Subjective stress was rated after each task, and participants indicated how difficult they found the task. They then sat quietly for a further 75 min, with additional blood samples at 45 and 75 min after tasks.

### 2.4 Measures

Body composition was assessed with a Tanita Body Composition Monitor (BC-418MA) from which body mass index (BMI) was derived. Anxiety over the past week was measured with the 7-item anxiety subscale of the Hospital Anxiety and Depression Scale (HADS) (Zigmond and Snaith, 1983), and positive affect with the positive affect subscale of the Positive and Negative Affect Scale (PANAS) (Watson et al., 1988). Scores on the anxiety scale could range from 0 to 21 and positive affect from 10 to 50, with higher ratings indicating greater anxiety or positive affect. Subjective stress and task difficulty were assessed on 7-point scales, with higher ratings indicating greater stress and perceived task difficulty.

Blood samples were drawn into EDTA tubes and immediately centrifuged at 2500 rpm for 10 min at room temperature. Plasma was removed and aliquoted into 0.5 ml portions and stored at -80° C until analysis. Plasma IL-6 was analysed with Quantikine high sensitivity two-site enzyme-linked immunosorbent assay (ELISA) (R&D Systems, Oxford, UK). The minimum limit of detection was between 0.016 and 0.110 pg/ml. IL-1Ra were analysed in duplicate using fluorescent-labelled capture antibody beads (Milliplex Human Cytokine/Chemokine kit, Millipore Corporation, US), and assayed using a Bio-Plex 200 Luminex system (Bio-Rad, Hemel Hempstead, UK). The limit of detection for IL-1Ra was 2.3 pg/ml. The mean intra-assay coefficient of variations (CVs) for IL-6 and IL-1Ra were 7.3% and 4.6%, and inter-assay CVs were 7.7% and 6% respectively.

### 2.5 Data reduction and statistical analysis

Systolic and diastolic BP and heart rate were averaged over the following 5 min periods: 25–30 min of the initial resting period (baseline), the 5 min of each behavioural task trial, min 15–20, 40–45 and min 70–75 following tasks. Differences in cardiovascular activity across task trials did not relate to pharmacological treatment, so the 3 task periods were averaged. Missing data for individual trials meant that 57 were included in analyses of IL-6 analyses, 59 in analyses of IL-1Ra, and 56 for BP and heart rate. Differences between treatment groups in background characteristics were analysed using analysis of variance with sex and treatment as between-person factors. Differences in baseline physiology were explored with analysis of covariance including age, BMI and smoking status as covariates. We analysed subjective stress, inflammatory and cardiovascular responses over the experimental session with repeated measures analysis of variance with sex and treatment as between-person factors, and trial as the within-person factor, again adjusting for age, BMI and smoking. Stress responses were analysed by computing change scores between baseline and task and recovery trials, including baseline levels as covariates. The majority of sessions (61.3%) took place in the morning, but the statistical results were unchanged when time of day was included as an additional covariate. Significant sex by trial interactions were followed by separate analyses of men and women. Statistical analyses were performed using SPSS version 24.0 (SPSS Inc., Chicago, Illinois, USA).

## 3 Results

Randomization of the 20 men and 44 women in this study resulted in equally sized propranolol and placebo groups, as detailed in Table 1. Participants were aged 23.37 years on average, and had healthy average body weights. The proportion of smokers was 12.5%, slightly lower than the national prevalence (15.8%). There were no significant sex or treatment differences in age, BMI, smoking status, anxiety or positive affect. All participants reported taking 7 capsules over the treatment period, except one who took 9 capsules since her stress session had to be rescheduled. Nine participants (14.8%) reported experiencing side effects that they attributed to the medication, but this number did not differ between propranolol and placebo groups (12.9% and 16.7% respectively). Oral contraception was being taken by 12 (27.3%) women, but was unrelated to the pattern of inflammatory or cardiovascular responses.

**Table 1:** Characteristics of study participants Means ± standard deviation or N (percent).

	Placebo		Propranolol	
	Men (n = 9)	Women (n = 23)	Men (11)	Women (21)
Age (years)	22.44 ± 3.5	22.13 ± 2.9	23.64 ± 2.9	25.00 ± 7.5
BMI	24.41 ± 2.7	22.73 ± 4.4	22.76 ± 2.5	22.53 ± 2.5
Current smokers	2 (22.2%)	2 (8.7%)	0 (0%)	4 (19.0%)
Anxiety	5.44 ± 3.2	5.68 ± 4.76	4.70 ± 3.3	4.86 ± 4.62
Positive affect	31.44 ± 5.7	35.00 ± 5.9	34.30 ± 3.7	33.67 ± 6.9

### 3.1 Baseline physiological activity

Physiological activity at baseline is summarized in Table 2. After adjustment of age, BMI and smoking status, there were no significant differences across treatment conditions in baseline IL-6 concentration, but levels were higher in women than men (*F*(1,51) = 7.16, *p* = 0.010). Baseline concentration of IL-1Ra was lower in the propranolol than placebo condition (*F*(1,52) = 6.71, *p* = 0.012). There was a main effect of sex in systolic BP (*F*(1,49) = 4.34, *p* = 0.042), together with a sex by treatment interaction (*F*(1,49) = 4.95, *p* = 0.031). The sex by treatment interaction was significant for diastolic BP as well (*F*(1,49) = 5.46, *p* = 0.024). As can be seen in Table 2, systolic and diastolic BP were lower in men treated with propranolol compared with placebo, with no difference in women. The analysis of baseline heart rate showed a significant main effect of treatment condition (*F*(1,49) = 11.73, *p* < 0.001), with heart rates being substantially lower in the propranolol than placebo groups.

**Table 2:** Baseline biomarker levels Means ± standard deviation adjusted for age, BMI, and smoking status.

	Placebo		Propranolol	
	Men	Women	Men	Women
IL-6, pg/ml N = 57	0.92 ± 0.45	0.91 ± 0.82	0.57 ± 0.19	0.89 ± 0.49
IL-1Ra, log pg/ml N = 59	6.10 ± 0.56	6.22 ± 0.37	5.80 ± 0.30	6.00 ± 0.33
Systolic BP, mmHg N = 56	116.2 ± 10.08	105.1 ± 10.30	105.5 ± 8.74	104.9 ± 10.21
Diastolic BP, mmHg N = 56	71.04 ± 10.74	65.32 ± 8.43	61.30 ± 7.53	64.86 ± 9.06
Heart rate, bpm N = 56	66.76 ± 7.82	72.08 ± 9.27	60.60 ± 9.45	61.92 ± 5.17

### 3.2 Subjective responses

Stress ratings increased significantly in response to tasks, from a baseline mean of 1.85 ± 0.96 to 3.80 ± 1.36, 3.82 ± 1.53, and 4.26 ± 1.66 after the speech, mirror tracing, and arithmetic tasks respectively (*F*(4,228) = 45.61, *p* < 0.001). There were no differences between treatment conditions or sexes in these responses. Similarly, ratings of task difficulty (averaging 5.21 ± 1.62 overall) did not vary between propranolol and placebo conditions (*p* = 0.32).

### 3.3 Acute inflammatory responses

**Fig. 1:**
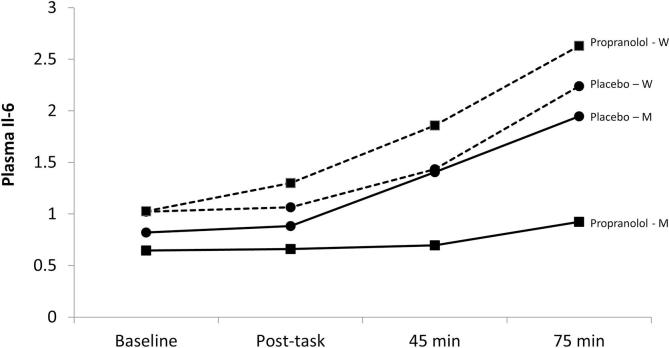
Mean concentration of plasma IL-6 (in pg/ml) adjusted for age, BMI and smoking status in samples obtained at baseline, immediately following tasks, and 45 min and 75 min following tasks in men (solid line) and women (dashed line). ■ = propranolol ● = placebo.

Analysis of plasma IL-6 concentrations over the session showed main effects for sex (*F*(1,50) = 4.67, *p* = 0.036) and trial (*F*(3,150) = 36.00, *p* < 0.001), together with a significant sex by trial interaction (*F*(3,150) = 3.92, *p* = 0.010). Men and women were subsequently analysed separately. As can be seen in Fig. 1, both men and women showed significant increases in IL-6 concentration following stress, with higher levels among women, after adjustment of age, BMI and smoking status. The treatment by trial interaction was significant for men but not women (*F*(3,48) = 8.10, *p* < 0.001), with a marked attenuation of IL-6 response to stress in men taking propranolol compared with placebo. This was confirmed in analyses of differences in IL-6 concentration at 45 min and 75 min after tasks compared with baseline, with smaller increases at both time points in the propranolol than placebo group (*F*(1,12) = 12.00, *p* = 0.005 and *F*(1, 12) = 15.28, *p* = 0.002 respectively). By contrast, there were no treatment effects among women.

The analysis of plasma IL1-Ra concentration showed a main effect of treatment condition (*F*(1, 51) = 4.55, *p* = 0.038), but no sex difference or trial effects. The lower levels of IL1-Ra already identified at baseline (Table 2) persisted throughout the experimental session, with no significant increases following stress.

### 3.4 Blood pressure and heart rate

**Fig. 2:**
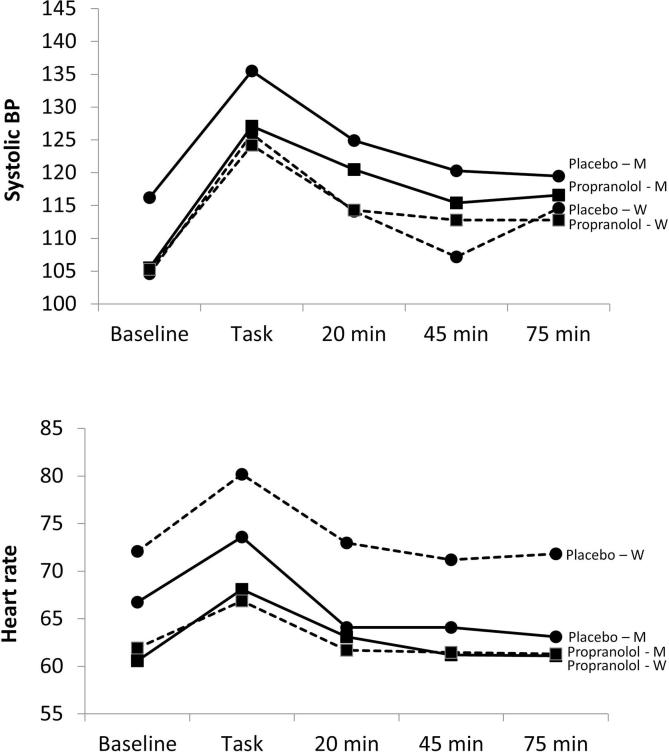
Mean systolic BP (mmHg, upper panel), and heart rate (bpm, lower panel) adjusted for age, BMI and smoking, in baseline, task trials, and 15–20 min, 40–45 min, and 70–75 min following tasks in men (solid line) and women (dashed line). ■ = propranolol ● = placebo.

Systolic BP responses across the session are shown in Fig. 2 (upper panel) adjusted for age, BMI and smoking status. Both groups showed marked increases in BP during tasks (*F*(4,204) = 54.30, *p* < 0.001), with partial recovery towards baseline over the post-task period. Systolic BP in men remained somewhat lower in the propranolol than placebo condition, but there were no significant differences in BP responses to tasks related to treatment in either men or women. A similar pattern was observed for diastolic BP (results not shown).

The analysis of heart rate showed main effects of trial and treatment condition (*F*(4,204) = 59.49 and *F*(1,52) = 18.25 respectively, both *p* < 0.001). Heart rate increased during tasks and reverted to baseline levels during the recovery period. The effect of propranolol was more marked in women than men, since there were significant treatment effects in all trials (*p* < 0.001), whereas differences between treatments were not present in the recovery period in men.

Associations between IL and 6 and systolic BP responses to stress were evaluated. Interestingly, systolic BP during tasks was correlated with IL-6 increases at 45 min (*r* = 0.80, *p* = 0.030) and 75 min (*r* = 0.79, *p* = 0.035), but only among men in the placebo condition. There were no associations among men in the propranolol condition, or among women in either treatment group.

## 4 Discussion

This placebo-controlled study evaluated the impact of 7 days treatment with the beta-blocker propranolol on acute inflammatory responses to mental stress. We hypothesised that propranolol would attenuate inflammatory and cardiovascular responses. The results provide only moderate support for this hypothesis. Treatment with propranolol resulted in attenuation of IL-6 responses to stress, but only in men and not women. Concentration of plasma IL-1Ra was reduced in the propranolol condition throughout the experiment, with no differences in stress response. Baseline heart rate was lower in the propranolol group among both men and women, while baseline systolic BP was reduced only among men. However, the intervention did not modify cardiovascular responses to stress in a clear way. For example, heart rate rose to a similar extent in response to stress in both placebo and propranolol conditions, although absolute levels were lower in the propranolol condition.

Mechanistic studies supported by animal research indicate a prominent role of sympathetic activation in stimulating expression of inflammatory cytokines in response to acute stress (Bierhaus et al., 2003, Sanders and Kavelaars, 2007, Watkins et al., 1999). Stress-induced expression of norepinephrine could increase pro-inflammatory cytokines for example by inducing NF- κB transcription. But a previous placebo-controlled study found no effect of propranolol on plasma IL-6 responses to the TSST in healthy volunteers (von Kanel et al., 2008). Our study has a similar design, involving 80 mg slow release propranolol daily, though this was administered for 7 instead of 5 days. The TSST was not used here, but the tasks (public speaking, mirror tracing and mental arithmetic) had similar properties, and were rated as stressful and difficult. The age of participants in the present study averaged around 25 years younger than those in the experiment conducted by von Kanel et al. (2008). It is not clear whether these factors accounted for our observation of an attenuation of IL-6 responses among men in the propranolol condition. We also showed that propranolol reduced baseline IL-1Ra, which was not the case for IL-6 suggesting that beta-adrenergic processes are involved in the production of IL-1Ra but not of basal IL-6. This result is in line with McNamee et al (2010) who showed that the beta-adrenoceptor agonist clenbuterol stimulates IL-1Ra, but not IL-6 in rats.

The reasons for the sex difference in the effects of propranolol are uncertain. We have no evidence that adherence to medication was greater in men than women. Estrogen influences the release of IL-6 (Rachon et al., 2002), but we found no differences in responses among women related to whether or not they were taking oral contraception, casting doubt on the relevance of reproductive hormone levels. Significantly larger plasma IL-6 responses to stress have previously been reported in women than men (Endrighi et al., 2016, Lockwood et al., 2016), and a similar effect was observed in this study (Fig. 1). Sex differences in central neurotransmitter and corticosteroid responses in relation to stress have been detailed (Bale and Epperson, 2015, Bekhbat and Neigh, 2018), but there has been limited focus on sex differences in sympathetic involvement in inflammatory responses. It is notable in the present study that plasma IL-6 and systolic BP responses to tasks were correlated among men in the placebo condition (an association abolished by propranolol), but not in women; this is consistent with the notion of sex differences in the role of the sympathetic nervous system. Evidence for sex differences in adrenergic receptor function is mixed, with some studies reporting decreased/increased or no difference on beta-adrenergic receptor responsiveness in normotensive individuals (Sherwood et al., 2017). Additionally, there is evidence for greater therapeutic effects of beta-blockers in men than women (Rosano et al., 2015). Consequently, differences in beta-adrenergic receptor responsiveness could partly explain the observed sex differences in our study.

Pharmacological blockade had limited effects on cardiovascular responses to stress. Although both BP and heart rate were reduced in the propranolol condition, this was a tonic effect that was already present at baseline, and stress responses were not affected. Previous studies have been inconsistent. A single dose of 80 mg propranolol administered 60 min before the TSST blocked heart rate responses in one study, but differences in systolic BP were not significant (Andrews and Pruessner, 2013). Similarly, no significant effect on BP stress reactions have been observed in other experiments (Alexander et al., 2007, Dreifus et al., 2014, Freyschuss et al., 1988). Cardiovascular responses to stress are highly conserved in many animal species so are likely to be tightly regulated.

In addition to sympathetic function, other mechanisms not mediated by noradrenergic receptors that may play a role in stress-induced inflammation and cardiovascular responses. Glucocorticoids play a major role in immune regulation, and are a core component of the physiological response to stress. Although they have profound immunosuppressive effects, glucocorticoids may also enhance inflammation, particularly at low doses (Cain and Cidlowski, 2017, Sapolsky et al., 2000). It has been argued that stress-induced increases in circulating glucocorticoids sensitise cells to pathogen-associated molecular patterns (PAMPs), danger-associated molecular patterns (DAMPs) and inflammatory cytokines (Dhabhar et al., 2012, Del Giudice and Gangestad, 2018). The parasympathetic nervous system is also implicated, since vagal stimulation inhibits production of IL-1β and IL-6 (Koopman et al., 2016). Heart rate variability is reduced during acute stress, with greater inhibition being associated with larger plasma IL-6 responses (Woody et al., 2017). These mechanisms may serve to regulate IL-6 responses to stress independently of adrenergic processes.

Participants’ hedonic state at the time of stress testing was evaluated by assessing anxiety and positive affect, while stress perceptions were monitored repeatedly across the session. There were no differences between treatment conditions in either men or women, although stress ratings did increase as anticipated during behavioural challenges. The purpose of these measures was to ensure that any differences in physiological responses were not a result of differences in mood. Likewise, since it is known that task engagement is associated with larger stress responses (Singer, 1974), we confirmed that task appraisals did not differ between experimental conditions. Thus differences between propranolol and placebo could not be attributed to subjective effects.

We did not observe any increase in IL-1Ra concentration following stress in this study. The impact of acute stress on IL-1Ra has been inconsistent (Marsland et al., 2017). Although IL-1Ra shows a robust association with a number of health risks including cardiovascular disease (Herder et al., 2017), its regulation is distinct from that involved in IL-6 expression (Dinarello, 2009, Schaper and Rose-John, 2015). One factor that may be relevant is the timing of blood samples following stress. For example, in one study IL-1Ra increases peaked at 90 min after stress (Rohleder et al., 2006), while another only documented responses at 120 min (Steptoe et al., 2001). The current study measured cytokine levels up to 75 min, and this may have been too short to demonstrate effects.

This study was carried out with young adults in good health, and we do not know whether similar findings would emerge with other groups. Post-menopausal women might not respond in the same way as the young women tested here because of differences in hormonal status. We did not use any objective measure of adherence to medication, but used pill counts, so it is conceivable that medication was not taken as instructed. The behavioural tasks generated marked increases in cardiovascular activity, subjective stress, and plasma IL-6 concentration, but other test protocols might have different effects. Salivary cortisol samples were collected during the study but proved uninformative, so have not been presented here. Strengths of the study include a rigorous double-blind design, detailed evaluation of cardiovascular activity using a continuous monitoring device, and assessment of subjective as well as biological responses.

In conclusion, we demonstrated that beta-adrenergic blockade with propranolol resulted in reductions in plasma IL-6 responses to acute mental stress, but only in men and not women. Future studies might measure the impact of beta-blockade on stress-induced inflammation in clinical samples of individuals with physical or mental health problems, and assess a broader range of pro and anti-inflammatory cytokines. The findings support a role of sympathetic nervous system activation in stimulating acute inflammatory cytokine responses to stress, but the reasons for the difference between men and women remain to be resolved.

## Conflicts of interest

The authors have no conflicts of interest to declare.

## Funding

This research was supported by the British Heart Foundation (RG/10/005/28296 and FS/13/40/30343).
